# Imaging myocardial scar in hypertrophic cardiomyopathy: advances in CMR and CT

**DOI:** 10.3389/fcvm.2025.1649728

**Published:** 2025-09-18

**Authors:** Matteo Sclafani, Chiara Perrotti, Luigi Salerno, Melwyn Luis Muthukkattil, Camilla Lustri, Gabriele Cristofari, Giulio Falasconi, Maria Beatrice Musumeci, Diego Penela, Andrea Saglietto, Agnese Iannaccone, Emanuele Barbato, Antonio Berruezo, Pietro Francia

**Affiliations:** ^1^ Royal Brompton and Harefield Hospitals, Guy’s and St Thomas’ NHS Foundation Trust, London, United Kingdom; ^2^ Cardiology Unit, Department of Clinical and Molecular Medicine, Sant’Andrea University Hospital, Sapienza University, Rome, Italy; ^3^ Arrhythmia Department, Teknon Heart Institute, Teknon Medical Center, Barcelona, Spain; ^4^ IRCCS Humanitas Research Hospital, Rozzano, Italy; ^5^ Division of Cardiology, Cardiovascular and Thoracic Department, “Citta Della Salute e Della Scienza” Hospital, Turin, Italy; ^6^ Department of Statistical Sciences “Paolo Fortunati”, University of Bologna, Bologna, Italy

**Keywords:** hypertrophic cardiomyopathy (HCM), cardiovascular magnetic resonance (CMR), cardiac computed tomography (CT), myocardial scar, late gadolinium enhancement

## Abstract

Myocardial scarring is a hallmark of hypertrophic cardiomyopathy (HCM) and a major driver of adverse outcomes, including sudden cardiac death and heart failure progression. The fibrotic substrate in HCM is complex, encompassing both replacement and interstitial fibrosis, often accompanied by myocardial disarray. Advanced cardiovascular imaging enables detailed scar characterization, which is crucial for risk stratification and personalized management. Cardiovascular magnetic resonance (CMR) is the gold standard for non-invasive fibrosis assessment. Techniques such as late gadolinium enhancement, myocardial mapping of T1 and T2 relaxation properties, and diffusion tensor imaging provide complementary insights into scar burden and architecture. Cardiac computed tomography (CT) is an emerging modality with increasing clinical relevance. Delayed iodine enhancement and CT-derived extracellular volume mapping offer a valuable alternative for scar assessment, particularly when CMR is contraindicated. This review highlights the role of multimodality imaging in assessing myocardial scar in HCM, with a focus on CMR and CT, and explores their clinical implications.

## Myocardial scarring in hypertrophic cardiomyopathy

Hypertrophic cardiomyopathy (HCM) is a prevalent inherited myocardial disease, affecting approximately 1 in 500 individuals in the general population (1, 2). It is characterized by left ventricular (LV) hypertrophy that occurs in the absence of systemic or cardiac conditions capable of inducing hemodynamic overload (3, 4). The hypertrophic myocardium in HCM exhibits a wide spectrum of structural alterations, both at the macroscopic and microscopic levels (5). In at least one-third of the myocardium, cardiomyocytes are hypertrophic and disorganized, displaying structural abnormalities in both shape and alignment, which is a phenomenon collectively referred to as myocardial disarray. While most pronounced in hypertrophic segments, disarray may also affect regions with normal wall thickness (5). The extracellular matrix in HCM is typically expanded and rich in glycogen, with widespread interstitial fibrosis that, in advanced stages, can lead to fibrotic replacement and the formation of myocardial scars (6). Additionally, microvascular abnormalities are a frequent histological finding, including medial hypertrophy, disorganized elastic fibres, and endothelial hyperplasia of the intramyocardial coronary arteries. These changes contribute to vessel wall thickening and luminal narrowing, leading to microvascular dysfunction and impaired perfusion reserve. The downstream consequences are myocardial ischemia, myocyte necrosis, and replacement fibrosis (6–9). The combination of myocyte disarray, extracellular matrix expansion, and microvascular dysfunction results in complex structural remodelling, with fibrosis -either interstitial or replacement- representing a pathological hallmark of the disease.

Myocardial scarring has been implicated in the most threatening outcomes of HCM, namely sudden cardiac death (SCD) and adverse LV remodelling (10). SCD is one of the most unpredictable and devastating complication of HCM, and it may be the initial manifestation of the disease. It occurs at an estimated annual incidence of 0.7% in unselected HCM cohorts (11) and disproportionately affects younger individuals, with a cumulative 5-year risk of approximately 8%–10% in paediatric patients (12). Ventricular arrhythmias, particularly ventricular tachycardia (VT) and fibrillation (VF) are the primary mechanisms underlying SCD (13), and myocardial scarring serves as a critical substrate for re-entrant circuits (14). Re-entry arrhythmias require the presence of a conduction barrier (anatomical or functional), two pathways with differing conduction velocities, a unidirectional block, and sufficient excitable myocardium. When these conditions are satisfied, electrical impulses may circulate continuously along a slow conduction pathway, reactivating previously recovered myocardium and leading to sustained VT (15). Dense fibrotic regions and anatomical structures act as non-conductive barriers, while myocardial disarray and interstitial fibrosis create a non-uniform conduction environment that promotes anisotropy and slowed conduction (16–18). Localized ischemia due to microvascular dysfunction contributes to electrical instability by creating zones of partial depolarization and further slowing of conduction, fostering arrhythmogenesis (18–21).

Adverse LV remodelling is another major disease progression pattern, occurring in approximately 15%–20% of HCM patients (22–24). It is defined by the superimposition of unfavourable structural changes upon the classic HCM phenotype (22). These changes include reduced LV ejection fraction (25), wall thinning (26), moderate-to-severe diastolic dysfunction (27, 28), marked left atrial enlargement (29), significant microvascular dysfunction (30), new-onset atrial fibrillation (31), spontaneous resolution of LV outflow tract obstruction (26, 32), and formation of LV apical aneurysms (33). Each of these features has been individually associated with poor outcomes in HCM cohorts. From a pathophysiological standpoint, adverse remodelling appears to result from a combination of chronic microvascular ischemia, cellular energy depletion, and myocyte apoptosis, ultimately leading to progressive myocyte loss and fibrotic replacement (24, 30, 34–38). The clinical manifestations of remodelling vary considerably, ranging from mild functional impairment to advanced heart failure (HF). End-stage HCM, the most extreme form, develops in approximately 5% of patients and is characterized by extensive fibrosis with either a hypokinetic-dilated phenotype, when systolic dysfunction predominates, or a hypokinetic-restrictive phenotype, marked by a small, stiff LV and severe diastolic impairment (4, 6, 25, 26, 35).

Together, SCD and HF, alongside thromboembolic complications, constitute the principal contributors to HCM-related mortality (25). Importantly, these outcomes are directly or indirectly related to the development of myocardial scar, which is a central prognostic marker. Consequently, the assessment of myocardial fibrosis has gained increasing importance in risk stratification and disease management.

Advances in cardiovascular imaging have greatly enhanced our ability to noninvasively characterize myocardial fibrosis in HCM. A multimodality approach including cardiovascular magnetic resonance (CMR) and cardiac computed tomography (CT) allows for comprehensive assessment of the extent, distribution, and nature of myocardial scarring, thereby informing prevention strategies and individualized risk stratification.

## Cardiovascular magnetic resonance

CMR is the gold-standard non-invasive imaging modality for the assessment of myocardial scar (39, 40). Owing to its excellent spatial resolution and superior tissue contrast, CMR enables comprehensive characterization of both replacement and interstitial fibrosis. In recent years, significant technological advancements have expanded the capabilities of CMR, allowing for an even more detailed evaluation of myocardial scarring and its prognostic implications.

This section will explore the main CMR techniques employed for scar characterization in HCM, outlining their technical principles, summarizing the current evidence in the literature, and discussing their clinical relevance.

### Late gadolinium enhancement

Late gadolinium enhancement (LGE) is the most established CMR technique for detecting replacement myocardial fibrosis (39). It involves the administration of a paramagnetic extracellular contrast agent (gadolinium), followed by the acquisition of T1-weighted images approximately 10–20 min after injection. This technique exploits the different kinetics of gadolinium distribution in healthy myocardium vs. scar tissue. Gadolinium accumulates in regions with expanded extracellular space due to necrosis or fibrosis, appearing as hyperintense areas on late-phase imaging, with signal intensity varying according to scar architecture (39).

LGE can be evaluated both qualitatively and quantitatively relative to total myocardial mass (39). Early investigations of LGE as a prognostic marker in HCM focused on total scar burden, which emerged as a strong predictor of SCD, adverse remodelling, and HF hospitalizations in several prospective unselected cohorts (16, 41–47). The largest study to date assessing the role of LGE in predicting SCD in HCM showed a significant increase in SCD events among patients with a high scar burden (≥15% of total myocardial mass), whereas no meaningful increase in SCD risk was observed in those with minimal LGE (1%–5%) compared to patients without detectable scar (16). Based on these findings, the American College of Cardiology and American Heart Association (ACC/AHA) included a scar burden threshold of 15% as a risk factor to be considered, particularly in patients lacking conventional clinical risk factors or when ICD implantation is uncertain (2). However, the use of scar burden alone has limited sensitivity, as numerous adverse events have been documented in patients with LGE ≤15% (7, 44, 45).

In HCM, LGE patterns demonstrate substantial heterogeneity in their extent, intensity, and distribution (48). A large prospective registry provided novel insights into this variability, showing that distinct LGE patterns are closely associated with specific morphological subtypes and sarcomere mutation status (49). This heterogeneity reflects the complex histopathological nature of HCM fibrosis, which differs from post-ischemic scars by comprising diffuse fibrosis interspersed with viable myocytes, rather than dense, localized subendocardial fibrosis within a specific coronary territory (7, 50–52). Such structural complexity creates a highly arrhythmogenic substrate, ideal for rapid-rate re-entrant VTs (15). These arrhythmias may progress to polymorphic VT or VF, driven by the distinct properties of the HCM myocardium—marked by sarcomeric disarray and heterogeneous, anisotropic conduction (53). CMR studies have demonstrated that areas of mild-to-intermediate LGE enhancement are more strongly associated with ventricular arrhythmias than intensely enhanced regions (54, 55). Moreover, in low- and intermediate-risk HCM patients, quantitative assessment of LGE heterogeneity (such as signal dispersion) has been shown to independently predict major arrhythmic events, even beyond total scar burden (48).

**Figure 1:**
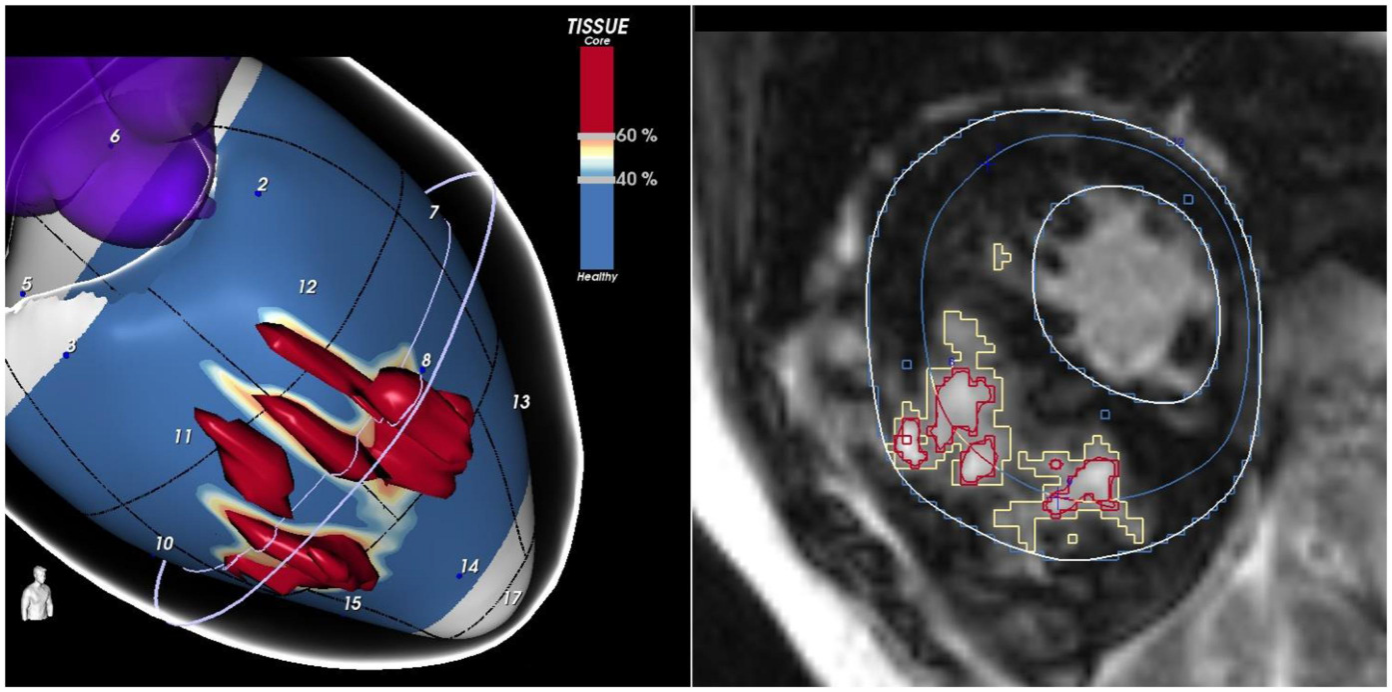
The figure shows a short-axis view of a CMR of a patient with HCM, highlighting an extensive scar involving the interventricular septum. LGE-CMR images were post-processed using ADAS 3D (Galgo Medical, Barcelona, Spain), creating nine concentric surface layers spanning from the endocardium to the epicardium of the left ventricular wall thickness, resulting in a 3D shell for each layer. Color-coded pixel signal intensity (PSI) maps were projected onto each shell. Hyper-enhanced areas were classified as the core zone, borderline zone (BZ), or healthy tissue using thresholds of 40 ± 5% and 60 ± 5% of the maximum PSI. The scar-dense core is coded in red, BZ is coded in orange and white, and healthy myocardium is coded in blue.

These findings support the notion that a qualitative evaluation of scar architecture, particularly LGE signal heterogeneity, may offer deeper insights into arrhythmic risk. However, conventional 2D LGE imaging has limitations in capturing the full complexity of the three-dimensional scar structure (48). To address this, advanced post-processing software have been developed to improve scar characterization (56, 57). These tools segment signal intensity at the pixel level to differentiate dense core fibrosis from diffuse border zone (BZ) fibrosis and reconstruct the data into 3D images (Figure 1). They identify corridors of BZ tissue surrounded by dense scars or anatomical barriers, which connect regions of viable myocardium, referred to as border zone channels (BZCs) (56). Functionally, BZCs represent slow-conducting pathways composed of excitable myocardium insulated by non-conductive fibrotic tissue serving as substrates for re-entrant VTs (56, 58). Recent CMR data demonstrated that the presence of BZCs is a strong independent predictor of ICD interventions for VT/VF in high-risk HCM patients (59).

### Myocardial mapping

Tissue characterization through myocardial mapping is a relatively recent advancement in CMR. By applying specific imaging sequences, this technique enables quantitative measurement of the longitudinal (T1) and transverse (T2 or T2*) relaxation times of each myocardial voxel (60). These values are then visually represented as color-coded parametric maps. This allows the detection of microstructural and biochemical myocardial tissue properties that are invisible to the naked eye, including the quantification of extracellular volume (ECV) (60). Both *in vivo* and *in vitro* studies have shown that ECV and T1 mapping are reliable surrogate markers of diffuse myocardial fibrosis, reflecting extracellular space expansion due to interstitial collagen deposition (61). By contrast, T2 mapping is a marker of myocardial oedema and inflammation (62).

A recent study in a large cohort of HCM patients demonstrated that interstitial fibrosis assessed via ECV and native T1 values was independently associated with cardiac death and provided incremental prognostic value beyond traditional clinical risk markers (63). Additional studies have further confirmed the adverse prognostic impact of interstitial fibrosis, linking it to unfavourable clinical features such as adverse remodelling (64), left ventricular diastolic dysfunction (65), and ventricular arrhythmias (66). Notably, interstitial fibrosis detected by T1 mapping and ECV is often present in myocardial segments without LGE and retains significant independent prognostic value (67). These findings suggest that interstitial fibrosis plays a central and independent role in the prognosis of patients with HCM.

A recent single-centre observational study in a large cohort of patients demonstrated that cardiovascular death or appropriate implantable cardioverter-defibrillator therapies occurred more frequently in individuals with elevated myocardial T2 values. These findings suggest a role of myocardial oedema in the pathophysiology of major adverse events and highlight T2 mapping as an additional parameter with potential value for prognostic stratification in HCM (68).

As such, mapping techniques have provided deeper insights into the role of interstitial fibrosis in HCM, particularly enhancing risk stratification in patients without LGE, traditionally considered low-risk (64, 69) However, combining mapping with LGE assessment has proven more effective than using either method separately, supporting the idea that LGE and mapping provide distinct but complementary information for a comprehensive evaluation of myocardial scar architecture (70).

Finally, a key advantage of tissue characterization by mapping is the ability to detect myocardial fibrosis sensitively without the need for contrast administration. Malek et al. demonstrated that automated machine learning models based on native (pre-contrast) T1 mapping can accurately identify myocardial fibrosis and closely align with LGE in HCM patients (71).

### Perfusion CMR

First-pass perfusion CMR during vasodilator stress is a well-established technique for detecting myocardial ischemia due to either obstructive epicardial coronary artery disease or microvascular dysfunction (72). Images are acquired during the first pass of an intravenous gadolinium bolus under pharmacological stress (most commonly adenosine or regadenoson). Perfusion can be evaluated qualitatively by visual inspection or quantitatively through the calculation of absolute myocardial blood flow (MBF, ml/min/g) and myocardial perfusion reserve (MPR, stress-to-rest MBF ratio) (73).

In HCM, microvascular dysfunction typically manifests as subendocardial perfusion defects with a near-circumferential distribution, often extending beyond a single coronary territory, and is associated with reduced MBF and MPR (74, 75). Several studies have demonstrated that inducible ischemia identified by CMR perfusion, whether assessed qualitatively or quantitatively, correlates with a greater burden of LGE, underscoring the central role of microvascular dysfunction in the development of replacement fibrosis (47, 76, 77). Furthermore, perfusion abnormalities have been associated with adverse clinical features, including ventricular arrhythmias and the formation of apical aneurysms, even independently of the overall fibrosis burden (76–79).

### Diffusion tensor CMR

Diffusion Tensor Imaging (DTI) is an advanced CMR technique that enables visualization of myocardial microstructure by mapping the three-dimensional diffusion of water molecules within the myocardium (80). By calculating fractional anisotropy (FA), cardiac DTI quantifies the directional variability of water diffusion: FA values approaching zero reflect isotropic diffusion (random, unrestricted movement), while values closer to one represent anisotropic diffusion (preferential movement along a single direction). Consequently, high FA values are observed in voxels with coherently aligned myocytes, whereas low FA values indicate disorganized or misaligned fibres due to myocardial disarray (18, 81).

DTI is currently the only non-invasive imaging modality capable of identifying myocardial disarray, thus offering significant diagnostic and prognostic potential in HCM (82–86). A recent study demonstrated that myocardial disarray detected via cardiac DTI independently correlates with ventricular arrhythmic risk, regardless of the degree of fibrosis or hypertrophy, suggesting a direct pro-arrhythmic role (18).

In addition, diffusion-weighted imaging (DWI), a simplified precursor of DTI, has emerged as a feasible alternative to native T1 mapping and ECV for the identification of interstitial fibrosis in HCM. A recent study demonstrated that the mean apparent diffusion coefficient (ADC) measured by DWI can identify areas of LGE with sensitivity and specificity comparable to that of T1 mapping and ECV quantification (87).

Despite its potential, DTI is still constrained by major technical challenges. Image acquisition is highly vulnerable to motion artifacts and requires complex, non-standardized protocols, while signal-to-noise ratio and reproducibility across vendors remain suboptimal (88, 89). Furthermore, the lack of universally accepted reference values for DTI-derived metrics, such as fractional anisotropy, limits clinical interpretation (90). As a result, DTI remains largely confined to research settings, and significant methodological refinements are needed before its integration into routine risk stratification in HCM.

### Radiomics

Radiomics is an emerging field in cardiovascular imaging that focuses on the extraction and analysis of high-dimensional quantitative features from medical images, effectively transforming visual data into mineable information (91, 92). The core principle of radiomics is that biomedical images contain a wealth of information that remains invisible to the human eye and is not captured through traditional qualitative interpretation (93). Radiomics seeks to uncover these hidden insights and derive novel biomarkers to improve diagnostic and prognostic accuracy, while reducing observer bias and subjectivity (94). Technically, radiomic analysis consists of several interdependent steps: image acquisition, raw data reconstruction, image pre-processing, segmentation, feature extraction, feature selection, and predictive model construction (95).

As previously discussed, scar heterogeneity in HCM is a critical prognostic factor that goes beyond simple quantitative assessment. Visual evaluation of scar heterogeneity, however, is often challenging and subject to considerable interobserver variability. Radiomics offers a promising solution by enabling a more objective and reproducible analysis. Recent studies in large, unselected cohorts of HCM patients have shown that LGE-based radiomic features reflecting scar heterogeneity are significant predictors of SCD and provide incremental prognostic value beyond current clinical risk models (96–98).

In addition to assessing LGE texture, radiomics has broader potential applications in scar characterization, including integration with myocardial mapping techniques (99–101). However, translation into clinical practice remains limited. Radiomic features are highly sensitive to variations in acquisition, segmentation, and pre-processing, raising concerns about reproducibility and generalizability (99, 102). The absence of standardized pipelines or validated multicenter datasets precludes routine use. At present, radiomics should be regarded as a promising but experimental approach, with clinical adoption contingent on methodological standardization and large-scale prospective validation.

### Serial CMR for monitoring scar evolution

Myocardial fibrosis in HCM is not a static phenomenon but a dynamic process that progresses over time, driving adverse ventricular remodeling and increasing arrhythmic risk (103). Longitudinal studies have shown that both the extent and heterogeneity of LGE frequently increase during follow-up, with progression rates influenced by clinical phenotype and patient profile (46, 47, 104). Importantly, fibrosis progression has been independently associated with higher rates of ventricular arrhythmias, sudden cardiac death, and the transition to end-stage remodeling, underscoring the prognostic significance of serial scar assessment (47).

In this context, repeat CMR examinations provide a unique opportunity to monitor scar evolution and refine risk stratification over time, particularly in patients who may not initially demonstrate extensive fibrosis. Serial CMR may be especially warranted in younger individuals, in those with intermediate risk profiles, or when clinical status changes. CMR thus emerges as a sensitive longitudinal biomarker of disease progression, enabling timely adjustment of preventive and therapeutic strategies in HCM.

## Computed tomography

Cardiac CT has shown growing potential in the assessment of myocardial fibrosis in recent years, driven by continuous improvements in spatial resolution and advanced post-processing techniques (105). As a result, CT has emerged as a viable alternative to CMR, particularly in patients for whom CMR is contraindicated or not available.

### Delayed iodine enhancement

Delayed Iodine Enhancement (DIE) represents the CT-based approach to direct assessment of myocardial fibrosis (106). This technique is conceptually analogous to LGE in CMR: gadolinium- and iodine-based contrast agents share similar kinetics, accumulating in regions with expanded extracellular space. Following the administration of iodinated contrast, late-phase images (typically acquired 5–10 min post-injection) allow for the identification of myocardial areas with altered contrast kinetics, indicative of scar tissue (107).

In HCM, several preliminary studies have shown that DIE enables the detection of late enhancement patterns topographically similar to those seen with CMR, predominantly located in the interventricular septum and at the ventricular insertion points (108). Comparative studies have confirmed this topographic concordance between DIE-CT and LGE-CMR, suggesting good sensitivity of the technique, especially in the presence of extensive or transmural scarring (109, 110).

However, DIE has several intrinsic limitations. CT offers lower contrast resolution compared to CMR, making it more challenging to detect subtle or patchy fibrosis. Moreover, fibrosis quantification is less standardized, although some studies have proposed attenuation thresholds in Hounsfield Units (HU) and semi-quantitative approaches (111, 112). Additional considerations include radiation exposure and the need for relatively high doses of iodinated contrast, which may be a concern particularly in younger patients or those with impaired renal function (106).

Despite these limitations, DIE-CT is an extremely valuable technique in patients who are not eligible for CMR or as a complementary tool when concurrent anatomical and coronary assessment is required (110). An interesting study in a small cohort of high-risk HCM patients with ICDs demonstrated that the myocardial fibrosis burden assessed by CT predicted ventricular fibrillation and ventricular tachycardia events (113). These findings were corroborated in an unselected group of HCM patients without coronary artery disease, where the presence of CT-detected scar was associated with higher rate of major adverse cardiovascular events (114). Larger, prospective studies are needed to validate the prognostic value of DIE in HCM.

### CT mapping

Contrast-enhanced CT techniques allow for the quantitative estimation of ECV. Technically, the principle is analogous to that of CMR, relying on HU attenuation measured before and after contrast administration (115). More recent technological advancements have further evolved CT mapping. Dual-energy CT or spectral CT systems acquire images at different energy levels, enabling the reconstruction of quantitative iodine distribution maps within the myocardium. Notably, these techniques do not require a pre-contrast scan, thereby reducing radiation exposure (116–118).

Several studies have demonstrated a good correlation between CT-derived and CMR-derived ECV in non-HCM patient cohorts (119–121), and Bandula et al. validated this approach histologically (122).

CT mapping potentially overcomes some limitations of conventional DIE, such as the subjectivity of visual thresholding for enhancement detection, particularly when integrated with emerging radiomics and deep learning post-processing techniques (115). However, clinical experience with CT mapping in HCM remains limited. To date, only one study has investigated the prognostic role of CT-derived ECV in a high-risk cohort of HCM patients with ICDs, revealing no significant association with the incidence of ventricular arrhythmias (123).

Overall, iodine mapping represents a promising frontier for the quantitative assessment of myocardial fibrosis in HCM. Further studies are needed to explore its clinical utility. On the other hand, widespread clinical adoption may be hindered by limited availability of dual-energy systems, increased post-processing complexity, and the need for optimized acquisition protocols.

## Conclusions

Myocardial scarring is a hallmark of HCM and a major driver of adverse outcomes, including SCD and HF progression. The fibrotic substrate in HCM is complex, encompassing both replacement and interstitial fibrosis, often accompanied by myocardial disarray. CMR is the gold-standard non-invasive imaging modality for myocardial scar evaluation. Indeed, techniques such as LGE, myocardial mapping, and DTI provide complementary insights into scar burden and architecture. CT is an emerging modality with increasing clinical relevance. DIE and CT-derived ECV mapping offer a valuable alternative for scar assessment, particularly when CMR is contraindicated.

Table 1 summarizes the applications of CMR and CT techniques in scar assessment.

**Table 1:** Applications of CMR and CT techniques in scar assessment.

Modality	Imaging technique	Scar characterization	Clinical significance
Cardiac magnetic resonance (CMR)	Late gadolinium enhancement (LGE)	Qualitative and quantitative assessment of replacement fibrosis	Strong prognostic marker for ventricular arrhythmias, sudden cardiac death and adverse remodelling (16)
	Myocardial mapping (native T1 and ECV)	Detection of interstitial fibrosis not visible with LGE technique	Marker of poor prognosis; useful for risk stratification in low-risk patients and when gadolinium is contraindicated (63)
	Diffusion tensor imaging (DTI)	Assessment of myocardial disarray	Independent arrhythmic prognostic role (18)
	Radiomics	Assessment of scar heterogeneity using multi-step automated quantitative analysis	Additional arrhythmic prognostic value beyond qualitative visual assessment of LGE (96)
Cardiac computer tomography (CT)	Delayed iodine enhancement (DIE)	Qualitative assessment of replacement fibrosis	Marker of poor prognosis. Useful alternative when MRI is contraindicated (113)
	CT myocardial mapping	Assessment of interstitial fibrosis	Emerging tool not yet specifically studied in HCM patients

LGE: Chan et al. (16).

Mapping: Wang et al. (63).

DTI: Ariga et al. (18).

Radiomics: Fahmy et al. (96).

DIE: Shiozaki et al. (113).

Cardiac CT and CMR offer complementary strengths in the multimodality imaging assessment of myocardial scar, each contributing synergistic information that enhances risk stratification and may inform individualized preventive strategies.
